# Stakeholder-driven development and implementation of CRICIT: an app to support high-quality data capture and protocol monitoring for outpatient clinical trials with vulnerable populations

**DOI:** 10.1017/cts.2023.609

**Published:** 2023-08-14

**Authors:** Katie Clark, Caleb Ruth, Kathryn A. Thomas, Katherine Dunham, Madelene Travis, Kristian Rivera-Santiago, Lauren Brinkely-Rubinstein, Emily Wang

**Affiliations:** 1 Department of Internal Medicine, SEICHE Center for Health and Justice, Yale School of Medicine, New Haven, CT, USA; 2 Research Allies LLC, Denver, CO, USA; 3 The Justice Collaboratory, Yale Law School, New Haven, CT, USA; 4 Department of Population Health Sciences, Duke University, Durham, NC, USA; 5 Information Technology Services, Yale University, New Haven, CT, USA

**Keywords:** Data science, data collection, database management systems, health information management, longitudinal studies, randomized controlled trial, electronic data capture (EDC), opioid use disorder, incarceration

## Abstract

**Introduction:**

Choosing an appropriate electronic data capture system (EDC) is a critical decision for all randomized controlled trials (RCT). In this paper, we document our process for developing and implementing an EDC for a multisite RCT evaluating the efficacy and implementation of an enhanced primary care model for individuals with opioid use disorder who are returning to the community from incarceration.

**Methods:**

Informed by the Knowledge-to-Action conceptual framework and user-centered design principles, we used Claris Filemaker software to design and implement CRICIT, a novel EDC that could meet the varied needs of the many stakeholders involved in our study.

**Results:**

CRICIT was deployed in May 2021 and has been continuously iterated and adapted since. CRICIT’s features include extensive participant tracking capabilities, site-specific adaptability, integrated randomization protocols, and the ability to generate both site-specific and study-wide summary reports.

**Conclusions:**

CRICIT is highly customizable, adaptable, and secure. Its implementation has enhanced the quality of the study’s data, increased fidelity to a complicated research protocol, and reduced research staff’s administrative burden. CRICIT and similar systems have the potential to streamline research activities and contribute to the efficient collection and utilization of clinical research data.

## Introduction

Developing and implementing randomized controlled trial (RCT) protocols can become increasingly complicated depending on the research methods, characteristics of the population, and features of the intervention. Although a detailed protocol is created during study development, unexpected challenges often arise during implementation. Sophisticated protocols help ensure high-quality science, but a protocol on paper is only as good as its fidelity during implementation.

In the digital age, there are many options for electronic data capture systems (EDCs) to manage RCTs [1,2]. As compared to paper-based systems, EDCs have been shown to expedite data availability, reduce clinical trial costs, ensure HIPAA compliance and data security, and improve data quality through real-time data monitoring and validation [3–7]. By allowing for data analyses to be conducted in real time, EDCs are particularly useful for translational research that involves rapid dissemination [4]. Robust EDCs reduce the risks of confidentiality breaches, protocol deviations, avoidable harm, and undue burden for RCT participants. Recent research has also discussed the benefits of user-centered design of EDCs, in which the system is specifically designed to meet the needs of the user, including increased user satisfaction and wider adoption [8]. Despite the importance of EDCs for scientifically rigorous research, Schmier and colleagues (2005) argued that study investigators often view EDCs as a secondary priority, after enrollment/recruitment and supply chain logistics [9].

EDCs are extremely diverse in terms of functions, capabilities, customization, and costs. Common EDC functions include data entry and validation, integration with other systems, randomization, data exports, and study reports [10]. Many factors including cost, functionality, design and the unique needs of a particular study make selecting the most appropriate EDC challenging. Some software is available for no extra cost if there is an institutional license, while others require commercial licensing. Often, there is not one existing EDC that has all of the functions needed for a complex RCT protocol, and thus researchers are forced to combine several software packages to meet the needs of their study. This can be especially challenging for multisite studies, in which the needs of each site may vary and must be considered when selecting an EDC.

In this paper, we document our process for developing and implementing a stakeholder-driven EDC for the management of an RCT that is evaluating the efficacy and implementation of an enhanced outpatient primary care model for individuals with opioid use disorder (OUD) who are returning to the community from incarceration. Our stakeholders include the individuals who are responsible for collecting data, overseeing the project, reporting to funders, and analyzing data. Stakeholders identified specific EDC features for the RCT that were not available in EDCs under consideration. These features included a robust participant tracking system, ability to allocate participants to treatment arms using an adaptive allocation algorithm, and ability to generate on-demand retention reports. After considering the needs of our stakeholders and evaluating multiple EDCs, our solution was to develop a custom EDC that could meet all of the complex needs of our RCT. Author KC devised the acronym “CRICIT” for our EDC, which stands for **C**linical **R**esearch **I**nformation **C**entralized database for **I**ntegrity and **T**racking. Here, we describe how we harnessed innovation from an interdisciplinary team of research staff, applied the principles of user-centered design, and utilized the Knowledge-to-Action framework to develop CRICIT.

## Material and methods

### Research overview

Starting in February 2020, we conducted an assessment to inform our development of an EDC for the Transitions Clinic Network: Post Incarceration Addiction Treatment, Healthcare, and Social Support (TCN PATHS) study. TCN PATHS is a NIH-funded hybrid type-1 effectiveness/implementation RCT assessing the effect of the Transitions Clinic Network’s (TCN) model of primary care on engagement in OUD treatment among individuals with OUD recently released from incarceration [11]. The core of the TCN model is the inclusion of community health workers into the primary healthcare team. TCN’s community health workers have a history of incarceration and have an active role in the patients care team, as they provide social support and address social determinants of health.

We are recruiting participants from five sites across the continental United States and Puerto Rico. Once enrolled, a participant is randomized to receive primary care from either a TCN program or a standard primary care program. From a translational lens, TCN PATHS focuses on clinical research in evaluating the effectiveness of the outpatient TCN primary care model and concurrently evaluates the implementation of the model.

### Conceptual framework: knowledge-to-action

**Figure 1.**
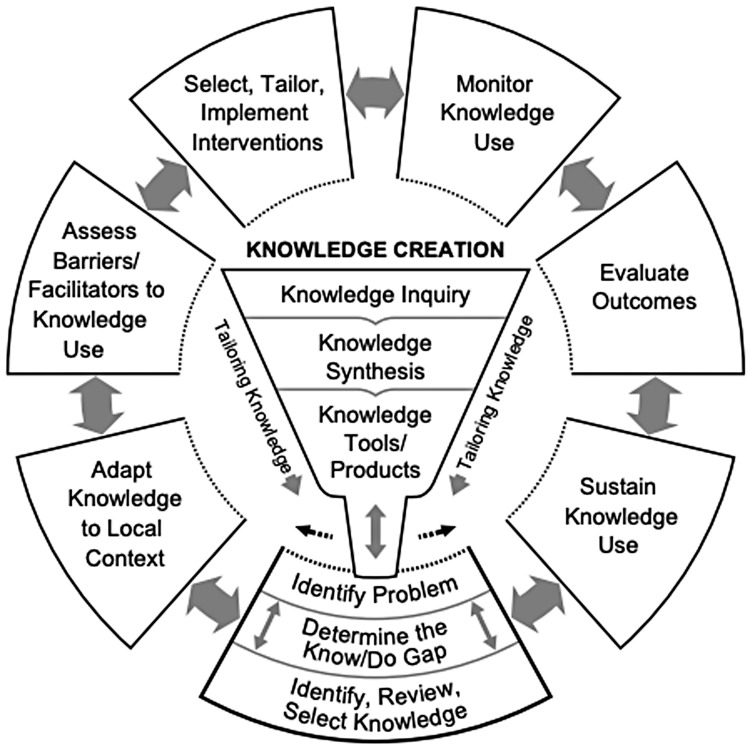
Knowledge-to-action framework. Graphical representation of the knowledge-to-action framework created and used with permission from “knowledge translation in health care: moving from evidence to practice” [13].

In developing CRICIT, we used the Knowledge-to-Action (KTA) conceptual framework, which is used to facilitate the knowledge translation process in health care [12,13]. KTA has two parts; at the core is the knowledge creation funnel and encompassing the funnel is the action cycle, as depicted in Fig. 1. Within the knowledge creation funnel, knowledge gained from primary studies and synthesizing knowledge on a particular topic are developed into guidelines or tools to assist in knowledge dissemination. As activities are conducted during this phase, the knowledge becomes more digestible and approachable to the end user and stakeholders. Once knowledge is transformed, the next step is to enter the action cycle by identifying, reviewing, and selecting the knowledge that is required to be implemented. From here, there are six additional steps in the action cycle: adapt to local context, assess barriers and facilitators, tailor interventions, monitor use, evaluate outcomes, and sustain use. Due to the complex nature of knowledge translation, these actions may be implemented in a nonlinear manner.

Although the KTA framework was developed to move knowledge into healthcare practice, we found KTA to be a helpful framework to guide the development and implementation of our EDC system. This framework was selected as common components of the software development life cycle (SDLC) can be mapped to the KTA domains, creating an interdisciplinary framework that leverages knowledge translation principles in the context of the SDLC [14]. Table 1 contains a mapping between common SDLC steps and the KTA domains, which demonstrates the alignment between the two.

**Table 1.** Framework mapping alignment between the common SDLC steps and the Knowledge-to-Action framework

Common SDLC Steps	Knowledge-to-Action Framework Domains
Identifying System Requirements	Knowledge Creation Funnel
Design and Development	Adapt Knowledge to Local Context Assess Barriers/Facilitators to Knowledge Use
Implementation, Testing	Select, Tailor, Implement Interventions
Deployment	Monitor Knowledge Use Evaluate Outcomes
Maintenance and Support	Sustain Knowledge Use

We created knowledge based on the original grant application and synthesized this into an implementation protocol to document what activities were required to accomplish the primary aims of the study. We facilitated conversations with study stakeholders to determine the gap between the required activities and the necessary actions to complete them. Working closely with stakeholders, we iteratively moved through both the knowledge generation funnel and the action cycle as we developed and implemented our EDC to include components of the original protocol and significant protocol adjustments that were made for numerous reasons, including adaptations due to COVID-19. During the design phase, we identified which aspects of the protocol needed to be adapted to local context, for example, access to site-specific consent forms and Spanish versions for our Puerto Rico site. The design phase also included identifying barriers and facilitators to protocol implementation and which design elements could be used to overcome barriers. During the implementing and testing phase, we programmed and tested various design elements and implemented the elements that provided the best solution to overcome barriers. Once testing was complete, we deployed our software then monitored use and evaluated outcomes by reviewing data, observing users in the system, and conducting group conversations to evaluate programming approaches. The maintenance and support phase included implementing new features and innovations to adapt to protocol changes and user needs.

### TCN PATHS EDC development

Using the implementation protocol that was developed during the knowledge creation phase, we identified stakeholders to be included in the EDC development process. Table 2 highlights the different roles of our stakeholders, how they interact with the EDC, and the key perspectives they contribute to the development process.

**Table 2.** Stakeholders’ key perspectives stakeholder’s interaction with electronic data capture systems (EDCs) and key perspectives

Role	Interact with EDCs	Key Perspectives
Research Staff	Screening, daily data entry, run due-for-study-visit reports, enrollment checklists, document interactions, and attempts to contact participants	Real-world implementation, identify “pain points” in protocol adherence, provide understanding of workflows and how this affects participants
Data Manager/Project Manager	Program the EDC, review back-end data for completeness and accuracy, develop reports to improve protocol compliance, develop reports to guide decision making, export data for analysis	Harmonizing needs of multiple stakeholders, collecting high-quality data with minimal staff burden, identifying micro and macro information for data-driven decision making
Co-Investigators	View reports, view individual participant charts, assess site specific and full project progress, use exported data for more sophisticated analysis, and/or merge with other datasets	Entire oversight of research goals, data required for funder reports, data required for in-depth analysis and merged datasets

Within the stakeholder roles, our research team spans multiple disciplines, which highlights different approaches and needs when developing the EDC. During the EDC development, we integrated perspectives and insights from multiple disciplines and fields of study including: bioethics, clinical research, computer science, data science, education, instructional and graphic design, medicine, public health, survey research, and statistics. Coinvestigators included statisticians, implementation scientists, and physicians who would participate in study data analysis. The data manager/project manager (KC) provided interdisciplinary leadership, leveraged the perspectives of the different roles and disciplines, and helped to establish common ground and identify specific components to meet the needs of the various EDC users. University software engineers and database administrators provided technical assistance regarding security and best practices in database design.

Through continuous conversations with stakeholders, we identified several foundational goals of our EDC. The system would need to support a complicated clinical research protocol. We use the term complicated, as the activities within a protocol are easily understood and are predictable (e.g., screening will result in a person being eligible or not), as opposed to the unpredictable and nonlinear nature of complex systems [15]. Although each component of the protocol is relatively simple, the synergy of multiple tasks and conditional procedures leads to a complicated protocol. System users would use the EDC to input and store various data about eligibility screening, participants enrolled, and activities to be completed, and to run summary reports that turn this data into information. Project management and coinvestigators would require a centralized system to oversee both site-specific and project-wide data. To the research staff, a centralized system meant that core study components (e.g., screening, randomization, enrollment, etc.) were contained within one system and did not require multiple software. Quality controls and data checks were essential to all stakeholders to ensure data integrity and adherence to the implementation protocol. Given that TCN PATHS is a longitudinal study with 13 study visits over the course of 12 months, stakeholders agreed that a robust participant tracking system would be an essential feature.

### CRICIT architecture

CRICIT was built using the Claris FileMaker Pro (FMP) software [16]. This software supports the rapid development of relational databases. Yale University has a FMP license and the Information Technology (IT) Department provides FMP server and administrative support. CRICIT is located on a HIPAA and electronic protected health information-compliant server that is also used by Yale New Haven Hospital’s Emergency Department. This server is backed up every hour. Prior to release, the database was reviewed by Yale University’s IT staff to ensure the system met security standards. CRICIT is enabled with encryption at rest to protect the system from a data breach and encryption in transit for network connections to the server. CRICIT was developed in the context of good clinical practice and complies with 21 CFR Part 11 including: limit system access and authority checks, utilization of an audit log, and system checks to assure appropriate sequencing of research activities [17]. Access to CRICIT is controlled by individual user accounts, passwords, and privilege sets. Research staff have a privilege set as “Data Entry,” which limits their capabilities to access and edit some data in contrast to the “Admin” privilege, which has full access to all data and editing privileges. The audit log documents the date and time at which changes were made to the data and the user responsible for the change.

CRICIT was programmed with system check techniques to assure appropriate sequencing of research activities. CRICIT uses data specific fields to prevent data errors and limit the use of open text fields. There are fields specific for dates, numbers, and text string dropdown menus, which will only allow users to input specific values. The data stored in these fields are used to trigger code that will make the next step in the research protocol available. This type of programming logic is used extensively in CRICIT to maintain fidelity to the protocol and assure correct sequence of activities.

**Figure 2.**
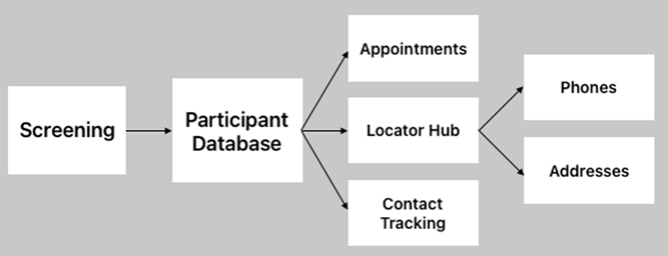
Database architecture. The rectangles represent data tables and a participant identification number links data across the tables.

CRICIT is a relational database, which allows data to be stored in separate tables, but because the data is related, a user is able to access data across tables. This is beneficial to our stakeholders in various ways. Our research staff is able to input and access data across tables for participant information (e.g., who is due for an appointment and if they are incarcerated) and our analytics team is able to access data within tables to conduct analysis (e.g., across the project how many participants completed an initial follow-up appointment). This allows a user to view both individual and aggregate data across various aspects of the protocol, as well as system metadata. The relational database design provides the structure and capabilities to support longitudinal studies. Fig. 2 is a graphical representation of the some of the key elements of CRICIT’s architecture.

### CRICIT implementation

The concept of CRICIT was developed in March of 2021 and the first version ready for users was deployed in May 2021. The CRICIT development team was led by the project manager, KC, and received extensive coding and graphic design support from one of the research staff, MT. Authors CR and KRS provided software development coaching and mentorship. The initial deployment included core protocol components, such as the ability to screen individuals, conduct the consent process, enroll participants, complete locator forms, track participant encounters, and schedule study visits.

In consultation with our stakeholders, we have adapted our protocol multiple times to adjust how the research environment has been affected by COVID-19. For example, initially the inclusion criteria stipulated only enrolling participants when they were still incarcerated; however, restrictions to research staff accessing these settings required us to expand our inclusion criteria to allow enrollment of individuals who were not currently incarcerated, but were released from incarceration in the past 30 days. Due to the rapid development environment of our software, we were able to adapt CRICIT to meet the protocol changes efficiently and with minimal lag between protocol decisions and software implementation.

We used the KTA framework to guide the initial CRICIT deployment, as well as managing the implementation of ongoing adaptations as we identified opportunities to streamline workflows in an applied research setting. Throughout the KTA cycle, we utilized user-centered design techniques, such as observing research staff using CRICIT and continuously seeking feedback, to inform software development [18,19]. For each protocol component that was incorporated into CRICIT, we began by assessing any adaptations that were necessary for each site; for example, some sites were collecting data for supplemental studies, while others were not.

We assessed barriers and facilitators to implementing the protocol, such as the case of identifying anchor dates for surveys. Our protocol required us to identify survey timeframes that begin 90 days prior or the date of the participant’s most recent study visit. Survey data are collected in the web-based software Qualtrics, and key data are passed from CRICIT into Qualtrics [20] to customize the questions for participants (e.g., “Since July 12th have you…”). Identifying the correct anchor date for a survey was a significant burden on the research staff and created a barrier to their efforts to comply with the protocol. We identified multiple coding interventions to address this barrier, and selected the intervention that would result in the least staff burden and highest data quality. We wrote an algorithm that would retrieve study visit dates, calculate the anchor date for the selected survey, then, using query strings, pass the information into Qualtrics which populated the accurate timeframes for a given survey. This solution improved adherence to the protocol, reduced research staff burden, and reduced data entry error.

**Figure 3.**
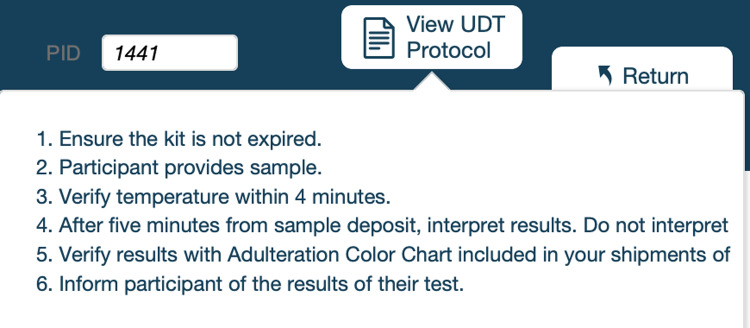
Informational buttons. Screenshot of informational buttons for urine drug testing (UDT).

As we implemented new components and features, we monitored and evaluated the backend data collection and user experience, making adjustments as needed. One of the ways we sustained research staff’s knowledge of the protocol was through the use of informational buttons. As shown in the urine drug testing (UDT) example in Fig. 3, these buttons allow research staff to access protocol details and definitions, thereby reinforcing information from the protocol. Additionally, CRICIT provides real-time status information on protocol adherence including screening, enrollment, and completed visit statistics.

We utilized multiple approaches to manage changes during our rapid development cycle to protect the integrity of our system and data. Two databases were created, a development database and a production database. The development database contains synthetic data for purposes of testing and development. The development database is where we programmed and tested new features to assure they were working as intended. Our software has a debugging function that is used to review code line-by-line to assure functionality and identify any errors. Once programming was thoroughly tested, we migrated the structural changes to the production database. Structural changes do not affect the data in the production database. Research staff were alerted to system updates and provided instructions on how to report features that were not working as intended. The project manager maintained a revision log that tracked reported problems, new feature requests, and how/when these issues were resolved. Additionally, the university IT Department backs up our system hourly, daily, and weekly, which would allow us to revert to a previous version, if needed.

## Results

CRICIT has been an essential tool for the research team to successfully implement a complicated study protocol. New features and software changes in CRICIT have kept pace with the protocol changes, including the integration of substudies. Barriers to efficient workflows have been solved through programming solutions, thus reducing burden on the research staff while maintaining exceptional data integrity in both quality and quantity. Importantly, these streamlined and centralized processes also benefit participants, ensuring that their data is stored in a secure and centralized location, reducing the time required from them during survey administration, and reducing errors and miscommunication from research staff. CRICIT has been customized to meet site-specific needs, including Spanish translation, and research staff only have access to their site-specific data. CRICIT has been instrumental to help the study team transform data into usable information.

**Figure 4.**
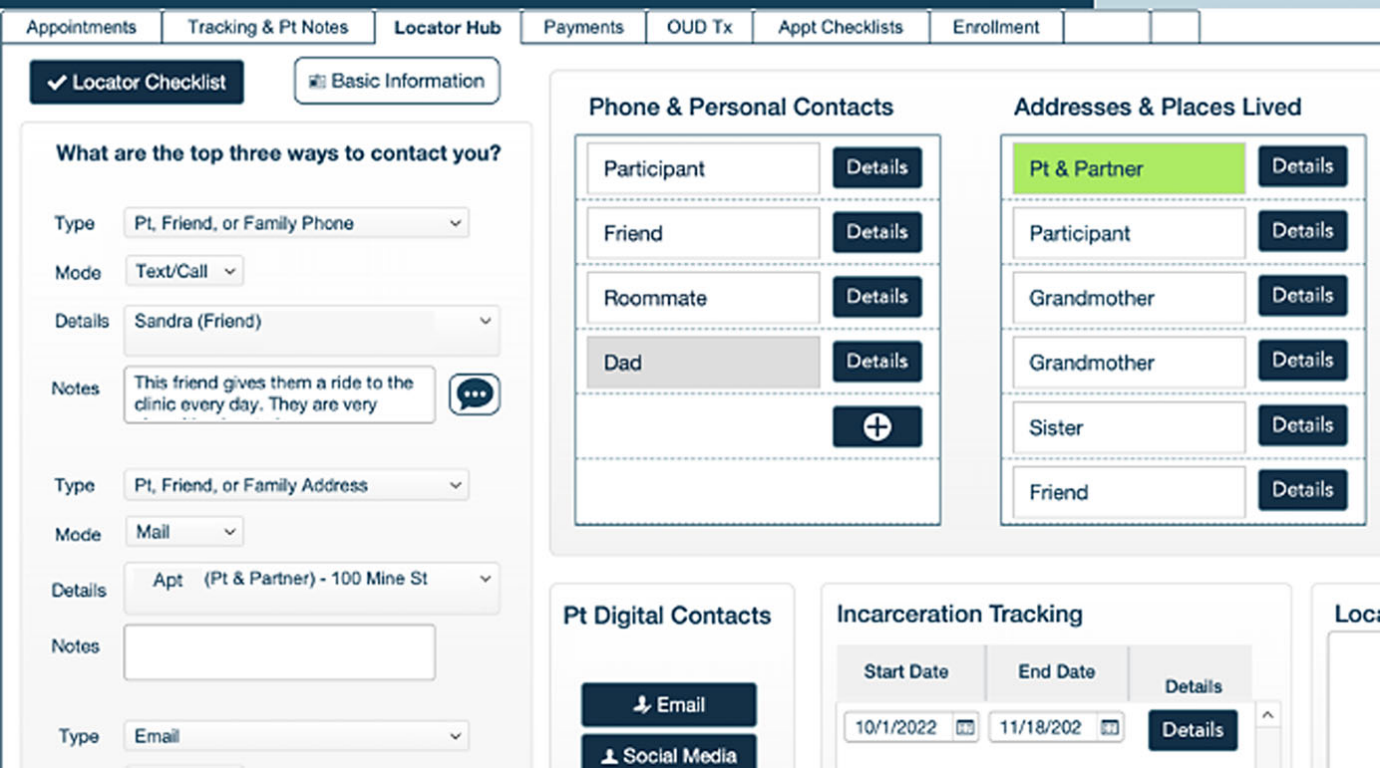
Locator hub. Screenshot of locator hub.

Throughout development, we optimized CRICIT to maximize the capabilities of the digital platform. Typically, longitudinal studies have a type of “locator form” that is used to collect participant location and contact information, such as phone numbers and relatives that could be contacted if research staff cannot directly communicate with the participant, as well as a history of past contact attempts. In previous studies, stakeholders have used paper forms or static electronic forms such as fillable PDFs, Word documents, or web-based forms. We re-envisioned this concept and developed the Locator Hub, a dynamic interactive module that facilitates capturing data about participants and their contacts and allows for high-quality and detailed tracking notes. Fig. 4 displays the Locator Hub. CRICIT auto-completes participant tracking records based on interaction with the phone portal. CRICIT has extensive participant tracking features to address the retention challenges inherent to longitudinal trials. The database’s comprehensive locator form allows participants to provide many different methods of contact. Additionally, users are able to keep detailed notes of their communications and communication attempts with participants.

We utilized the Pocock and Simon covariate-adaptive sequential minimization method to allocate participants to treatment arms [21]. Initially, the allocation algorithm was programmed into an Access database and stored on a desktop computer. Research staff had to connect to a virtual private network, remote into the computer, open Access, enter duplicate data from CRICIT into Access, run the allocation algorithm program, then log out and return to CRICIT to enter the arm to which the participant was allocated. Approximately twice per month, the Access computer would need to be restarted or other in-person troubleshooting, which was difficult to coordinate during COVID-19 restrictions. To improve workflow, we integrated the allocation algorithm into CRICIT, which runs when a participant is enrolled in the study. By integrating this feature within CRICIT, staff workload and participant wait times were reduced, and our enrollment workflow is no longer dependent on an unreliable system.

Development of an organized and attractive graphical user interface, informational pop-ups, and visual conditional formatting make CRICIT intuitive for the user and minimizes training. Informational pop-ups provide protocol details, instructions, and other helpful information that users can access in the moment without having to leave CRICIT. These pop-ups reduce errors, reduce frustration, and improve adherence to the protocol. Visual conditional formatting guides the user on correct data entry by highlighting required fields and sending error messages if a value is outside of an acceptable range.

### Research staff

CRICIT has enabled research staff to track study participants through the entire course of the study participation. Specifically, staff can screen and enroll participants, manage appointments and payments, and document completion of study benchmarks. CRICIT allows research staff to document COVID-19 screenings, urine drug testing results, and OUD treatment. Research staff are also able to run reports to identify participants who are due for appointments, reminder calls, and check-in calls. Reports can also identify participants who have missing data in their chart. Key data quality features designed to reduce human error and increase efficiency include automated data entry, dropdown lists, error messages for outlying values, and conditional formatting to highlight missing data. CRICIT allows for time-efficient data collection and entry, thereby minimizing research staff’s cognitive burden and respecting participants’ time during study appointments.

Research staff report CRICIT has significant advantages over other software they have used to conduct other clinical trials. They appreciate the “one-stop-shop” functionality of CRICIT and having key components to conduct the research contained within one software program. Research staff have enjoyed the experience of identifying a problem, working with the team to identify a solution, and then seeing the problem resolved and the solution implemented. They find the graphic design visually appealing and the user interface easy to navigate.

### Project manager

The project manager was able to continually update CRICIT to better serve the evolving needs of the project and the research staff. They developed reports to monitor participant retention and research staff’s use of the system, and to perform data quality checks. To ensure data integrity, users are also able to log and request necessary data corrections throughout CRICIT. These requests populate a log for data cleaning.

**Figure 5.**
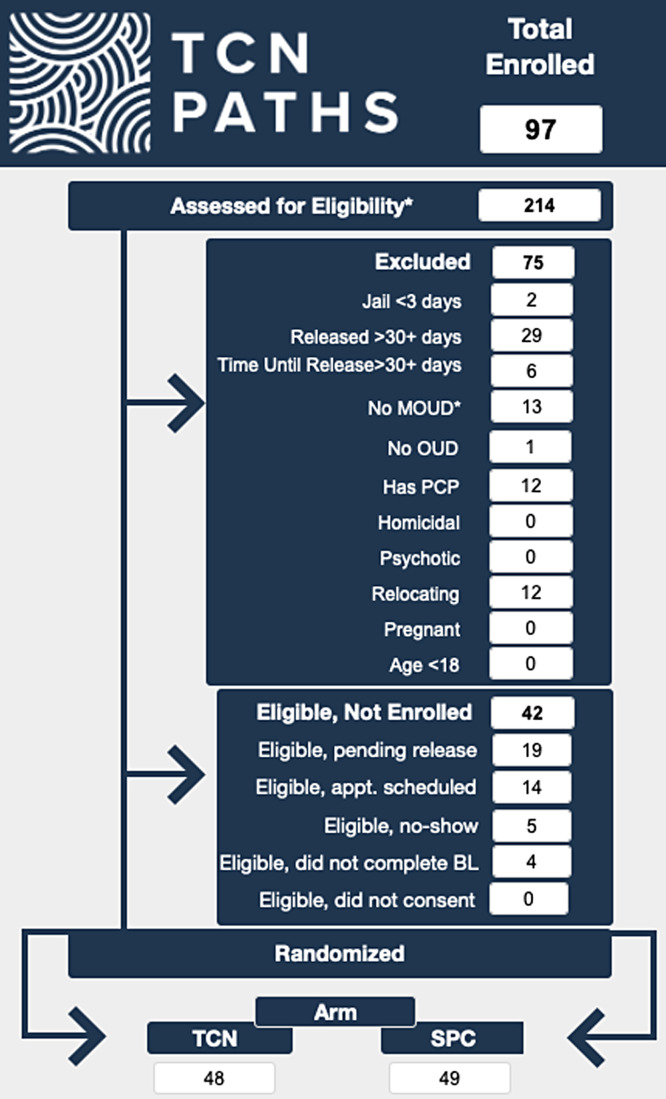
Reports. Screenshot of eligibility flow diagram.

The project manager regularly receives requests for information about the study from coinvestigators and funders. We created various reports in CRICIT including an enrollment dashboard, a retention report, and a CONSORT flow diagram (see Fig. 5) that are instantly available with real-time data. We are also able to quickly generate deidentified datasets for our funder. These reports are coded on the backend, and when a user pushes the button to generate the report, the code consistently runs to produce the report with the currently available data. Only qualified programmers have access to the code, which ensures the quality and reproducibility of these reports. The ease of access to data in various forms, including raw data sets, dashboards, and reports, facilitates identifying trends in data collection and provides information to support data-driven decision making.

From a leadership perspective, the project manager utilized shared decision-making techniques to collaborate with the research staff on CRICIT implementation and improvements. The project manager integrated the research staff in all aspects of CRICIT updates and sought their feedback after a new feature was implemented. When research staff request a system change or new report, the project manager was able to implement these requests, which fosters a positive group dynamic.

### Co-investigators

Coinvestigators are end-users of the data that are collected and synthesized in CRICIT. The Principal Investigator uses CRICIT-generated reports to lead meetings and provide collaborators with updates. Reports, such as the retention report and CONSORT flow diagram, are designed to be visually appealing and are readily available to share with collaborators and funders. The designed reports in CRICIT provide efficient access to data and assist the end-user to help translate data into understandable information. Coinvestigators rely on data from CRICIT to understand the study’s progress and make data-driven decisions. Enrollment data have been used to make decisions about the rate of enrollment at specific sites, adjust enrollment goals, and predict dates when enrollment goals may be achieved.

Due to the rapid development environment of CRICIT, coinvestigators have been able to adjust the protocol to adapt to changes such as challenges presented by COVID-19 and the integration of sub-studies. When changes were made to the protocol, we were able to quickly make changes in CRICIT to support implementation. We were able to implement specific COVID-19 screening protocols that followed the requirements for each site. We were also able to integrate sub-studies within the TCN PATHS broader research. This has allowed the Principal Investigator to support early career faculty and provide them with access to enrolled participants and well-trained research staff. CRICIT was programmed so only sites that are participating in sub-studies will see and have access to pertinent information.

## Discussion

Research staff are often responsible for the majority of data collection and participant interaction in clinical trials. Their work involves adhering to complicated research protocols, which are not adequately supported by off-the-shelf EDC. Developing a custom EDC provided our research team with a tool to support fidelity to the research protocol, high-quality data collection, and efficient data utilization in a single platform. Our EDC supported our participant-facing research staff while also meeting the data export needs for our data analysts and statisticians.

Characteristics of quality clinical trials include adherence to the study protocol, balanced study arms for analysis, and participant retention [22–24]. We designed CRICIT to support these priorities as well as reduce administrative burden on research staff, strengthen data integrity, and minimize the need for data cleaning. CRICIT’s organizational and visual design help research staff identify what data collection points are required per our protocol. Informational pop-ups reinforce learning the protocol and help research staff avoid errors. Co-investigators and project managers can use CRICIT reports to gain insight into overall study progress.

For longitudinal studies, participant retention is paramount to quality research [25,26]. CRICIT supports retention efforts in multiple ways. CRICIT includes a comprehensive Locator Hub and tracking system that allows research staff to enter data about participant contacts and efforts to communicate with participants. The real-time retention reports provide a dashboard of survey completion rates, which is helpful for our stakeholders to understand how the full study and individual sites are performing regarding participant retention and protocol adherence.

Selecting an EDC is a critical decision for an RCT team. Many researchers may default to an EDC that they have used in prior studies or use a system that is recommended due to institutional licensing agreements. Our novel EDC provides the security, customization, and simple data export capabilities as other popular systems, such as REDCap. Additionally, CRICIT has incorporated a robust participant tracking system and real-time participant retention and CONSORT flow diagram reports. Not all RCT will have complicated protocols that require a custom EDC such as CRICIT; however, for those that do, we have demonstrated that developing a system with these comprehensive features is a feasible and worthwhile endeavor. Researchers exploring the possibility of developing a custom EDC are encouraged to consult with professionals in regulatory compliance, information security, data management, and other disciplines as needed to identify system requirements and available software platforms given the specifications of their study.

### Challenges

Overall, developing and implementing CRICIT has been a positive experience for our team. During this process, we did encounter some challenges. At project launch, we did not anticipate building a custom EDC system. This created a human resource challenge as KC and MT added learning new software tools and EDC development, in addition to their previously established responsibilities. Each protocol change also triggered a CRICIT update, which would require time to implement, test, and deploy. The second challenge we experienced is logistics to access the software across a multisite team. Staff were required to download the software. Most research staff possessed university-managed laptops and did not have administrative privileges. Because of this, they were required to meet with their IT department to assist with the installation of the software, which delayed access to CRICIT during the onboarding process. The other significant challenge we experienced was that individuals were not familiar with the software and/or had misconceptions about the software’s limitations and functionality. We overcame this challenge by providing in depth information and conducting functional demonstrations of CRICIT, including training videos accessible by research staff. The software itself has some current limitations. The built-in graphing functions are not intuitive and web access via a web browser is challenging. Data visualization and web access have not been identified by our stakeholders as essential to our research implementation.

### Plans for future enhancements

As we continue to use CRICIT for the remainder of the TCN PATHS study, our planned future enhancements are focused on participant retention. Currently, if primary contact information is not successful in locating participants, the research staff move through a progressive series of activities in an attempt to locate participants. One of these activities is visiting the publicly accessible websites of carceral facilities and searching inmate information to determine if a participant has been reincarcerated. We plan to automate this activity within CRICIT by scanning publicly available data sources and matching this data with our enrolled participant database. Team members have previously implemented this web scraping technique in an EDC similar to CRICIT by utilizing an Application Programming Interface (API) call and a concise code block. This would benefit our clinical research in multiple ways. First, this would alleviate research staff from having to manually conduct this activity and reduce data lookup and entry errors. Second, research staff would be alerted when a participant was incarcerated, thus reducing the time spent on trying to locate the participant. Given the significant amount and quality of data that will be collected in CRICIT throughout the course of the study, we have an opportunity to quantify our experience with participant retention activities and contribute to this topic in the clinical trial literature.

As previously mentioned, our survey data are collected in Qualtrics. While this software has many beneficial attributes, there are several challenges including a labor-intensive workflow to access partially completed surveys, inefficient access to recorded survey responses, and limited control of visual design. Incorporating survey data collection directly in CRICIT would introduce a more efficient data workflow for both research staff and the project manager. This would also allow our team to access and fully customize the graphical user interface and implement survey visual design best practices. These improvements would yield a better user experience for participants and research staff and improve data quality and data availability.

### Dissemination

We have provided CRICIT demonstrations to numerous groups including fellow NIH Justice Community Opioid Innovations Network colleagues, FileMaker for Researchers User Group, and other teams who are exploring EDC options [27,28]. During these demonstrations, we highlighted the functionality of CRICIT and discussed the process of developing an EDC with a multidisciplinary team. For presentations with fellow developers, we sought feedback and collectively solved challenges.

CRICIT was developed specifically for the TCN PATHS RCT protocol. If we had developed CRICIT for a different protocol with different stakeholders, the result would have been a different system. While RCTs likely have common features, such as tracking participant scheduling, recording payments, and collecting contact information, these features should be tailored to the protocol, participant needs, and stakeholders. We have spoken with colleagues who have implemented their own version of CRICIT [29]. One programmer without previous FMP experience was able to implement their EDC in about two months, which was similar to our experience and timeline. This adds to the evidence that developing a custom EDC is accessible to study teams with a broad range of coding and technical experience and is not likely to create extensive barriers to timely implementation [30,31]. We hope to collaborate with researchers from various disciplines who are or would like to begin managing their studies with custom EDC. This collective work could result in developing code and modules that would support efficient implementation of new systems, harnessing new technology, and fostering opportunities for interdisciplinary team science.

Based on our experience developing CRICIT, we have identified process recommendations for multidisciplinary research teams considering the development of a custom EDC. Stakeholder involvement is critical to the successful development of custom EDC. Our key stakeholders included various individuals, including participant-facing research staff, IT and database security experts, research professionals and investigators, implementation specialists, data pipeline personnel, and those with user design experience. Each stakeholder provides important perspectives that are utilized to develop systems that are secure and support various team members’ contribution to the research. Interdisciplinary leadership is essential to provide a vision for innovation, manage efforts to translate research objectives into implementation protocols, and guide custom EDC development. Teams should design systems that accommodate protocol modifications and establish plans to monitor subsequent changes.

## Conclusion

Custom EDC development with a user-centered and protocol-focused design approach enhanced the informativeness and quality of the clinical trial data. The KTA framework guided this development and was useful to translate the research protocol into real-life use. CRICIT’s customizability allowed us to integrate protocol components directly into the EDC and adapt to emerging challenges. In doing so, we were able to enhance the quality of the study’s data and reduce research staff’s administrative burden.

We found the resources required to develop and implement a custom EDC that were a worthwhile investment given the advantages for participants, research staff, and investigators. CRICIT and similar systems have the potential to streamline research activities and contribute to the efficient collection and utilization of clinical research data.
